# Exploring neural question generation for formal pragmatics: Data set and model evaluation

**DOI:** 10.3389/frai.2022.966013

**Published:** 2022-10-31

**Authors:** Kordula De Kuthy, Madeeswaran Kannan, Haemanth Santhi Ponnusamy, Detmar Meurers

**Affiliations:** Department of Linguistics, University of Tübingen, Tübingen, Germany

**Keywords:** question generation, German, question-answer dataset, Questions under Discussion, discourse analysis, neural network

## Abstract

We provide the first openly-available German QUestion-Answer Congruence Corpus (QUACC), designed for the task of sentence-based question generation with question-answer congruence. Based on this corpus, we establish suitable baselines for question generation, comparing systems of very different nature. Question generation is an interesting challenge in particular for current neural network architectures given that it combines aspects of language meaning and forms in complex ways. The systems have to generate question phrases appropriately linking to the meaning of the envisaged answer phrases, and they have to learn to generate well-formed questions using the source. We show that our QUACC corpus is well-suited to investigate the performance of various neural models and gain insights about the specific error sources.

## 1. Introduction

Questions are at the center of various research strands, both in modern theoretical linguistics and in computational linguistics. In theoretical linguistics research mostly focuses on the special structure of questions and how and in which way meaning is conveyed by questions. The main interest in questions in current research in computational linguistics evolved around the task of question answering, as for example required by dialogue systems, and in the field of question generation. Much of the current research targets questions and question generation (QG) under a Question Answering (QA) perspective where the task is to produce a question that is related to some information given in a text passage. The focus here is thus on the functional link between the question and the information that answers it. Several data sets, such as the Stanford Question Answering Dataset (SQuAD) (Rajpurkar et al., 2016), the Conversational Question Answering dataset (CoQA) (Reddy et al., 2019), and the Question Answering in Contex dataset (QuAC) (Choi et al., 2018) have been created for this task, providing sets of questions and the text passages that contain the requested information.

Complementing QG in the prominent QA context, there are other strands of QG research that aim at generating questions that can be answered by a sentence as given in the text, putting a premium on question-answer congruence. This includes QG work in the educational application domain, where the perspective of the question is supposed to reflect the perspective of the author of a given text passage that the student is supposed to learn about (Heilman and Smith, 2010; Heilman, 2011; Rus et al., 2012). And it includes work for which the relation between the question and the answer sentence as expressed in the text plays a crucial role which includes research interested in discourse. An early example of research investigating the role of discourse structure for question generation is Agarwal et al. (2011). They identify discourse relations in a text as cues motivating the generation of a question and then formulate questions that can be answered by the sentences with those discourse relations, while ensuring direct question answer congruence. In a related vein, approaches making use of so-called Questions under Discussions (QUDs) to identify the information structure of a sentence in a given discourse also rely on such a direct relationship between question and answer. This task of question generation with question-answer congruence requires the design of different data sets than the above mentioned QA based set such as SQuAD. It requires data sets that contain question-answer pairs with explicit question answer congruence. First approaches exploring question generation under the perspective of question answer congruence are presented in the work of De Kuthy et al. (2020) and Kannan et al. (2021). Based on a newly created data set several word-based, character, and subword seq2seq models are trained and tested that successfully generate questions satisfying question answer congruence, i.e., questions that can be answered with the sentences given in the input.

The goal of this article is to establish/explore QA congruence based question generation in sufficient detail both in terms of an appropriate data set and in an in-depth evaluation of suitable methods for this task. However, to the best of our knowledge, there are currently no openly available QA-congruence corpora that permit such an enterprise. Available QA data sets, such as SQuAD, do not contain enough examples that meet the requirement of direct question-answer congruence between a question and an answer sentence. To address this lack of data, this paper introduces QUACC, the Question Answer Congruence Corpus, a corpus of 5.3 millions question-answer pairs obtained from a German newspaper corpus, designed explicitly for the task of QG with direct question answer congruence. A first version of this corpus was presented in De Kuthy et al. (2020). While they focused on the quality of the neural question generation models, they did not further investigate the quality of the newly created data set. Since neural models are very sensitive to the quality of the data, some of the quality issues observed by De Kuthy et al. (2020), such as generation of incorrect question words, seem to be related to the errors in the data set. We therefore developed method to clean the original QUACC data set which will be discussed in this article. This cleaned QUACC resource allows for a variety of data-driven experimentation and opens the way for more research in the area of question generation and beyond.

In terms of suitable methods, we explore different architectures for question generation with question-answer congruence for authentic German data. Proceeding sentence by sentence through the text, the task is to automatically generate a question for a given sentence and a given answer phrase. In principle, transformation rules can transparently express the potential types of question-answer pairs, e.g., a *who* question asking for the subject of a sentence, or a *when* question asking for a temporal adverbial. But while the relationship between the question phrase and the answer phrase can sufficiently be expressed by such transformation rules, the selection of the proper question phrase, the identification and removal of the answer phrase, and the reformulation of the sentence into question form and word order depends on a complex interplay of factors. Neural architectures with their ability to adapt to multiple patterns required by a specific task thus seem to be a much more robust approach for question generation in the context of question answer congruence.

To test performance and trade-offs between various neural architectures using character-level, subword-level, and word-level representations in the context of question generation for question-answer congruence, we further advanced the German question generation task proposed by De Kuthy et al. (2020), aimed at generating a Question under Discussion for each sentence in a discourse. The required question-answer congruence with the meaning and form requirements this entails, together with the relative morpho-syntactic richness and partially flexible word order of the German language make it an interesting experimental setting for exploring the potential advantages of several neural architectures, such as models based on character and subword representations. The structure of this article is as follows: In chapter 2, we present the already mentioned requirement of question-answer congruence in more detail, and we discuss examples illustrating the particular challenges arising when trying to generate questions under the perspective of strict question-answer congruence. In chapter 3, we present the creation of the German QUACC corpus and discuss detailed characteristics, such as ratio of question words etc, in the corpus. Chapter 4 then introduces the topic of questions generation, first giving an overview of existing approaches and then presenting all our neural approaches that were trained and tested on the different versions of the QUACC cropus. Finally, in chapter 5 we provide a comprehensive evaluation of all our neural models, both in terms of calculating BLEU scores and in terms of an in-depth human evaluation. The paper closes with a short outlook on other neural architectures that have been shown to be suitable for generation task, such as transformer-based architectures, and other evaluation methods that could be explored for the evaluation of the quality of neural QG models.

Our contributions in this paper thus are two-fold. First, we provide the first openly-available German QUACC corpus, aiming at introducing the task of sentence-based question generations with question-answer congruence. Second, we establish suitable baselines for question generation, comparing systems of very different nature.

## 2. The challenges of generating questions with question answer congruence

Why is a special data set containing question-sentence pairs, where each sentence is a complete answer to the preceding question, i.e., there is direct question-answer congruence, of interest at all?

As mentioned in the introduction, the research typically targets QG in the context of Question Answering, where the task is to generate a question that is related to the information in a given paragraph. The QA task ensures a general functional link between the question and the meaning of the passage that answers it. The data sets designed for such question answering/generation provide paragraph-level contexts for each question that span multiple sentences or even multiple passages. Note that the question here is related to the information expressed in the text passage, not to the way in which this information is structured and expressed in the text.

**Figure 1:**
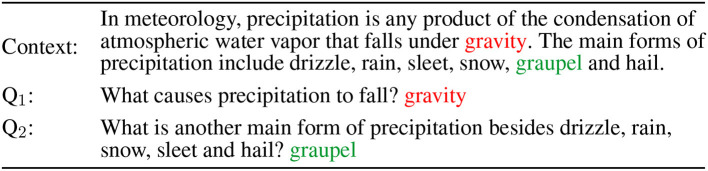
Example question-answer pairs from the SQuAD dataset (Rajpurkar et al., 2016).

The example from the SQuAD data set shown in Figure 1 presents a typical example in this domain. The first question pertains to the first sentence of the passage. While the concept *gravity* mentioned in that sentence is needed to answer the question, the question cannot be answered using the first sentence as such. For the second question, the information needed to answer the question is expressed in a sentence that is more in line with the question, but still falls short of the so-called question-answer congruence (Stechow, 1990; Sugawara, 2016) required for the sentence to serve as a direct answer to the question.

Complementing questions in the prominent QA context, there are other strands of QG research that aim at generating questions that can be answered by a sentence as given in the text, putting a premium on question-answer congruence. This includes, as mentioned in the introduction, QG work in the educational application domain, where the perspective of the question is supposed to reflect the perspective of the author of a given text passage that the student is supposed to learn about (Heilman and Smith, 2010; Heilman, 2011; Rus et al., 2012). Recent work under this perspective includes Stasaski et al. (2021), who propose a neural question generation architecture for the generation of cause-and-effect questions. They extract cause and effect relations from text, which are then used as answers for the neural question generation, aiming at direct question-answer congruence.

Another strand of work where question-sentence pairs with direct question answer congruence play a crucial role is the research interested in discourse. In particular, approaches making use of so-called Questions under Discussion (QUDs) to identify the information structure of a sentence in a given discourse rely on such a direct relationship between question and answer. The intuitive idea that the informative part of an utterance is that part that answers the current QUD is also mentioned in corpus-based research attempting to analyze the information structure of naturally occurring data (Ritz et al., 2008; Calhoun et al., 2010). Yet, these approaches were only rewarded with limited success in terms of inter-annotator agreement, arguably because the task of identifying QUDs was not made explicit. More recently, Ziai and Meurers (2014) and De Kuthy et al. (2016) showed that for data collected in task contexts including explicit questions, such as answers to reading comprehension questions, focus annotation becomes more reliable. The explicit question context enables experts and non-experts to reach substantial agreement in the annotation of discourse functions such as focus. In addition, automated annotation of information structure becomes feasible when explicit questions are given (Ziai and Meurers, 2018). Bridging the gap from corpora already containing explicit questions to the analysis of any type of authentic language data, Riester et al. (2018) spell out a discourse annotation approach in which explicit pragmatic principles define how a QUD can be formulated for every assertion expressed by a text. De Kuthy et al. (2018) and De Kuthy et al. (2019) show that in corpora that are manually annotated with explicit QUDs, information structure concepts such as focus and topic can be annotated with higher inter-annotator agreement than in previous work only implicitly making use of the idea of QUDs. While explicitly annotating corpora with QUDs appears to be a key for reliable manual or automatic annotation of information and discourse structure, in all of the above approaches it is a complex manual step. Exploring how to at least partially automate this complex enterprise of enriching corpora with suitable questions is the main objective of the work presented in De Kuthy et al. (2020), Kannan et al. (2021). They trained word, character and subword seq2seq models successfully generating questions that satisfy question answer congruence, i.e., questions that can be answered with the sentences given in the input. This work also openly discusses where the particular challenges of generating question with strict question answer congruence mostly occur. For example, Kannan et al. (2021) observe that the seq2seq architecture used for QG quite often fails to select the correct question words and the correct word order for the generated question. Another problem are rare or unknown words that have to be predicted. In most neural generation architectures, words are the basic input and output tokens. Pretrained word embeddings are used to initialize the token embedding matrix and generally a fixed vocabulary (e.g., the 150k most frequent words) is used for both input and output sequences. With a restricted vocabulary, given the Zipfian distribution of words in language use, in any authentic corpus material serving as input there are likely to be rare or unknown words that are not part of the fixed vocabulary and therefore cannot be predicted in the output layer, the generated question. This indeed is a major issue mentioned for the question generation approach of De Kuthy et al. (2020). To overcome this problem, they implemented an *ad-hoc* post-processing step: After a question has been generated, it is checked for markers indicating the places where an out-of-vocabulary token appears. A heuristic then tries to identify that missing word in the source sentence and insert it in the right place of the output.

**Figure 2:**
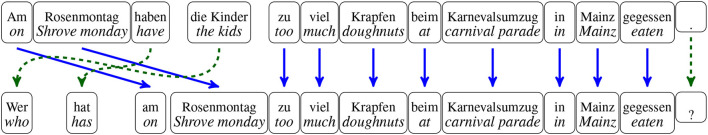
An example showing identical words in source sentence and question (with solid blue links) and the question word and subject-verb agreement requiring changes in the question formulation (dashed green relation).

When we conceptually consider the task of question generation from source sentences with the requirement of question answer congruence, this is a problem that should not arise—after all, the source sentence is explicitly provided and the words in the question to be generated can be selected from that source material, to which the question words, which can be drawn from a fixed set of language expressions for a given language, need to be added. So the task of generating a question based on a given sentence conceptually consists of two subtasks: (i) Identifying the material that is identical between source sentence and question and can simply be copied over, and (ii) predicting the new material appearing in the question, in particular the correct question words. This is illustrated by the sentence-question pair in Figure 2. In that example, the specialized carnival terminology, *Karnevalsumzug* and *Rosenmontag*, are typical rare words, and the use of the city name *Mainz* illustrates the occurrence of named entities.

**Figure 3:**
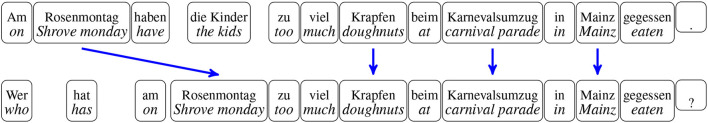
Example illustrating minimal identification of rare words and named entities in support of QG.

For the mentioned example, Figure 3 identifies the minimal case, i.e., the rare or unknown words that should be copied, whereas other words can or need to be generated to fit the output context, such as the question word *wer (who)* and the subject-verb agreement that needs to be adjusted from plural *haben (have)* to singular *hat (has)*. Kannan et al. (2021) show in a detailed analysis of the generated questions of their neural subword-based QG models, that indeed the models have high attention weights for the marked answer phrase and the verb in the source sentence and that most other tokens are just copied over as-is from the source sentence to the output question.

## 3. Data

The above overview of the challenges related to question generation with question answer congruence highlights the need for a QA data set especially tailored toward this task in order to successfully create and evaluate QG methods. The creation of such a suitable training data sets is challenging, mainly due to the sparsity of naturally occurring data already containing enough explicit question answer pairs. In the general line of research approaching QG in the context of question answering (QA), QA corpora such as SQuAD (Rajpurkar et al., 2016), Coqa (Reddy et al., 2019), or Quac (Choi et al., 2018) are typically used to train and evaluate neural QG models. However, such corpora are not well-suited given our goal of generating questions with direct question answer congruence. As illustrated above, these corpora provide a paragraph-level context for each question, where the question is related to the information encoded in the paragraph, not to the way this information is structured and presented in a sentence. So Q-A-Congruence between the question and a sentence that answers it is not ensured. For research like ours that focuses on the direct link between a question and the sentence providing the answer, corpus data that does not ensure Q-A-Congruence is insufficient.

In addition, there is only a very limited number of resources for languages other than English. The few existing, mostly multilingual, parallel data sets such as XQUAD (Artetxe et al., 2019) and MLQA (Lewis et al., 2019) are evaluation data sets of very limited size. Another option would be to automatically translate a corpus or design a neural model architecture to jointly translate, align and generate questions (Carrino et al., 2019). While this is potentially promising, it substantially increases complexity and potentially reduces performance due to translation error propagation.

Thus, to create a suitable QA data set, we need to create a corpus fulfilling three desiderata: (a) containing naturally-occurring data of German, (b) featuring a well-balanced set of questions of different types, and (c) ensuring direct question answer congruence between each sentence and question.

### 3.1. Corpus creation

For the creation of a QA corpus without any preexisting data resources, we started out with the only question related German resource at hand, the rule-based question generation system of Kolditz (2015), which he kindly made available to us. This system made it possible to create a suitable QA corpus from scratch. Creating such a corpus required a large, authentic German text source. for which we chose the German newspaper *Die Tageszeitung* (TAZ, https://taz.de) which in the science edition is available in XML format and has also been used for the German TüBa-D/Z treebank (Telljohann et al., 2004).

For the original QA answer corpus established in De Kuthy et al. (2020), 450K individual TAZ articles from years 1995 to 2001 were extracted using *Beautiful Soup 4* (https://crummy.com/software/BeautifulSoup) and were tokenized and segmented using *spaCy*'s (https://spacy.io) de_core_news_sm model. Sentences with fewer than four tokens, not starting with an uppercase letter, or not ending with a period or exclamation mark were filtered out. The resulting 5.46 million sentences were fed into an updated version of the rule-based QG of Kolditz (2015), producing a corpus of 5.24 million triples of the form < sentence, question, the answer phrase in the sentence given the question>. This German QUACC (Question Answer Congruence Corpus) includes questions with 43 different types of question phrases. The most common types of answer phrases, for which the rule-based system can generate questions, are NP subjects and objects, and many types of PP objects, as well as various types of adverbial modifiers. Furthermore, the set of potential answer phrase also includes (finite and non-finite) clausal constituents. The examples (1) and (2) show two typical examples for question answer pairs in the QUACC corpus with the answer phrase marked in bold.


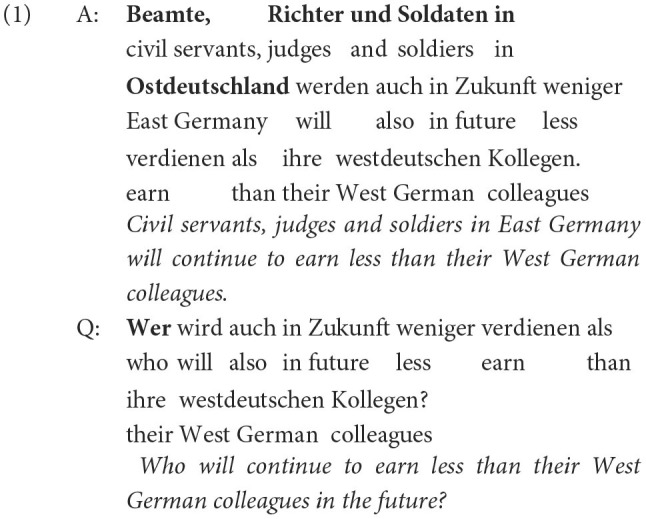



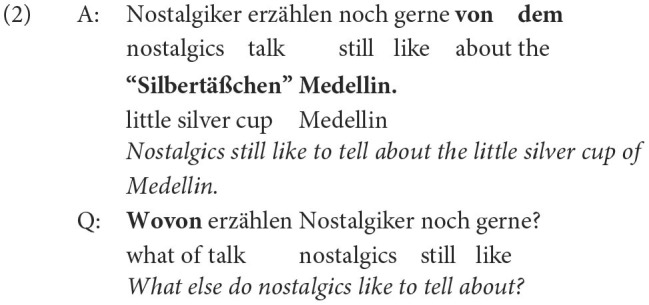


In (1), the complex subject NP shown in bold is replaced by the matching question word *Wer* (“who”), and the question is generated with adjusted agreement morphology on the finite verb (*werde*→*wird*). In (2), the PP object in bold is replaced by the question word *Wovon* (“what of”), and the question appropriately integrates the originally sentence-initial phrase (*Nostalgiker*).

For any given sentence, the rule-based system identifies all possible answer phrases and generates one question for each answer phrase. The example in (3) illustrates how many types of questions, based on the chosen answer phrases the transformation rules can in principle produce for a given sentence.


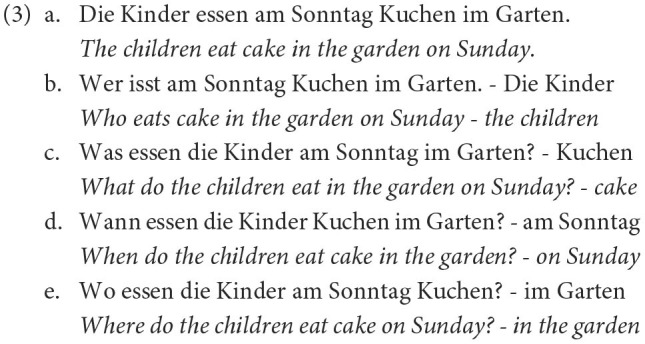


For the final QUACC data, individual < sentence, question, the answer phrase> triples were compiled for each answer phrase and matching question for a given sentence. This means that the sentences in the QUACC can occur multiple times, but each in a different S-Q-A triple.

The generated question answer pairs in the QUACC corpus all satisfy the requirement of question answer congruence and as shown in De Kuthy et al. (2020), this data set is a good source for training and testing of question generation approaches. De Kuthy et al. (2020), Kannan et al. (2021) trained word, character and subword seq2seq models successfully generating questions that satisfy question answer congruence. But, although in De Kuthy et al. (2020) it is observed, that the rule-based system is not very robust and does make errors when generating questions [De Kuthy et al. (2020) report, that 37% of the questions produced by the rule-based system for a sample of 500 sentences from the TAZ newspaper corpus are in fact not well-formed questions] the quality of the 5.4 questions in the QUACC data set is never further investigated. This is mostly due to the fact that the models presented in De Kuthy et al. (2020) and Kannan et al. (2021) produce questions of such high quality (both in terms of BLEU scores and in terms of human evaluation) that it seems the models picked up patterns of well-formed questions in German despite being trained on a noisy data set. All the models do show very high BLEU scores, which is measured as n-gram overlap between questions produced by the neural models and rule-based questions, with scores showing up to 90 % overlap. but this high n-gram overlap does not say anything about the well-formedness of the questions. The manual evaluation, however, reveals that that only around 60 % can be considered well-formed questions. This shows that the models picked up the patterns found in the rule-based questions, but they also picked up patterns that resulted in the production of non-well-formed questions. To further investige whether these errors are the result of noisy data or problems of the neural models itself, we here present a method how to best create a clean QUACC data set mostly consisting of well-formed question-answer pairs.

### 3.2. Cleaning the data set

Since the cleaned QUACC data set should be of similar size as the unclean data set, it is clear that a method based on a pure manual evaluation of the questions to identify the well-formed question-answer pairs is not feasible. We therefore decided to create a manually labeled gold data set and then train a classifier on these gold-labeled data that can then determine for our large QUACC set whether a given question is well-formed and exhibits question answer congruence or not. Since we want to create a clean question answer data set with grammatical questions meeting QA congruence between the question and the answer for each question-sentence pair, the quality criterion for the manual evaluation involved both grammatical well-formedness of a question and meaningful question-answer congruence.

For the creation of the manually labeled gold data, in a first round 8 German native speakers labeled sets of 2,000 question answer pairs each, in a second round 9 German native speakers labeled sets of 3,000 question answer pairs. In both rounds, the annotators were given the following evaluation criteria which were presented in form of written annotation guidelines before the labeling process.

- die Frage: Ist die Frage grammatikalisch korrekt und würde ich diese als MuttersprachlerIn des Deutschen so formulieren?

*the question: Is the question grammatically correct and would I formulate it this way as a native speaker of German?*

- das Frage-Satz Paar: Wird die Frage von dem dazugehörigen Satz als Ganzes beantwortet?

*the question-sentence pair: Is the question answered by the associated sentence as a whole?*

For the labeling itself, the randomly selected question answer pairs from the QUACC corpus were presented online in the doccano tool (Nakayama et al., 2018), and each question-answer pair had to be assigned one label with the following definitions specified in the annotation guidelines:

- Die Fragen müssen grammatisch korrekt sein und auch von der Bedeutung her Sinn machen, nur dann werden sie als “perfekt” bewertet.

*The questions must be grammatically correct and also make sense in terms of meaning, only then will they be marked as “perfect”*.

- Wenn eine Frage wohlgeformt ist und auch inhaltlich Sinn ergibt, nur das Fragewort nicht zu dem Antwortsatz passt, dann soll das Label “w-wort” ausgewählt werden.

*If a question is well-formed and also makes sense in terms of content, but the question word does not match the answer sentence, then the label “w-word” should be selected*.

- Ist die Frage nicht wohlgeformt, das Fragewort passt aber trotzdem zu dem Satz, wird das Label “fehlerhaft” vergeben.

*If the question is not well-formed, but the question word still fits the sentence, the label “incorrect” is given*.

- Wenn beides der Fall ist, die Frage ist fehlerhaft und das Fragewort ist nicht das passende, werden beide Labels vergeben, “w-wort” und “fehlerhaft”.

*If both is the case, the question is incorrect and the question word is not the appropriate one, both labels are assigned, “w-word” and “incorrect”*.

In the annotation guidelines, explicit examples for each label were given. As an example for a question with a non-matching question word (i.e., not showing proper question-answer congruence), which should be assigned the label *w-wort*, the following example was given:


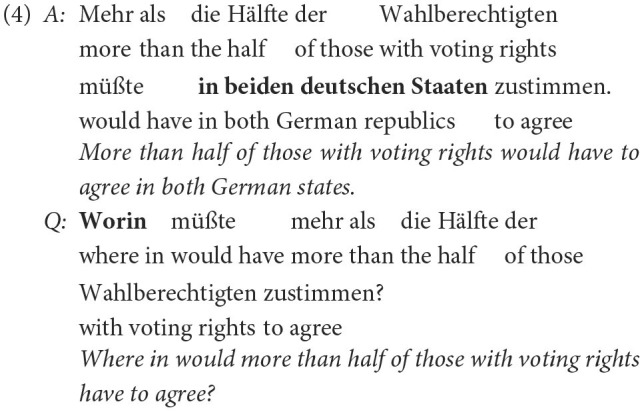


The correct question word in the question in (4) would be *wo* (“where”) instead of *worin* (“where in”). The question is nevertheless a well-formed question in German. But without the correct question word, there is no question answer congruence.

For the case which contains two errors, i.e., a non-matching question word and a grammaticality error, the following example was provided:


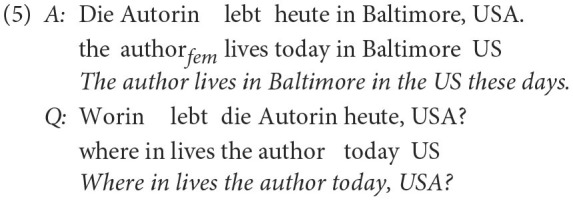


In addition to the incorrect question word ( *worin* (“where in”) instead of *wo* (“where”)) the question in (5) is also not well-formed, since part of the answer phrase *Baltimore, USA* is repeated in the question. So this question would need to be labeled both with *w-wort* and with the label *fehlerhaft* (“incorrect”).

Before the labeling, all annotators labeled a test set consisting of 200 question answer pairs, the labels were compared to make sure that all annotators showed sufficient inter-annotator agreement.

The resulting gold-label corpus consists of 35.750 labeled question answer answer phrase triplets, 50 % of the question-answer pairs were labeled “perfect” (i.e., grammatically well-formed and with question answer congruence), 11.6% were labeled with *w-wort*, 36% were labeled *incorrect*, 2.4% received both labels, *w-wort* and *incorrect*. This gold-labeled QA set can be made available upon request.

### 3.3. Training a neural classifier

The next step is to create a cleaned QUACC corpus with the help of the gold labeled QA answer pairs. This should be done automatically with the help of a classifier that identifies those QA pairs in the original corpus that are not well-formed and do not meet QA congruence. To train such a neural classifier on the gold labeled question-answer pairs, we chose the Transformer-based (Vaswani et al., 2017) Electa architecture (Clark et al., 2020). In contrast to traditional generative Transformer models such as BERT (Devlin et al., 2018) and its derivatives, Electa utilizes an adversarial architecture to train a generator network and a discriminator network. The former functions as a masked language model that outputs the probability of predicting a specific token for each masked position in the input. The outputs of this network are fed into the discriminator network, which in turn predicts if the token at each position belongs to the original sentence or was replaced by the generator.

To expedite the process of training the model, we chose an existing German Electra model^1^ from the HuggingFace Transformers Python library (Wolf et al., 2020) to serve as our classifier model's base. The model was pre-trained on combination of the following web corpora: German Common Crawl corpus^2^ (2019-09) (Wenzek et al., 2020), German Wikipedia Articles (2020-07), German Subtitles, and German news articles from 2018.

After re-purposing the final layers of the model for binary classification, we fine-tuned it on 30,400 samples from our annotated gold data set for 10 epochs. As inputs to the model, both the sentence and its corresponding question were concatenated in a pre-processing step with a special separator([SEP]) meta-token and then tokenized. Batch size was set to 16, and the warm-up steps for the learning rate scheduler was set to 500. Training was performed with the Adam optimizer (Loshchilov and Hutter, 2019) and a weight decay factor of 0.01.

At the end of each training epoch, the model was evaluated by calculating the F1-score of its predictions on 1,789 samples from the development set. Finally, the model with the highest score was chosen as the final model. The predictions of our final model on a held-out test set of 3,576 samples resulted in an F1-score of 0.843 (*P* = 0.803/*R* = 0.88). Since our data set was human-annotated with a high level of inter-annotator agreement, a further qualitative analysis of the results was not performed.

Upon classifying our original QUACC corpus with this model, we were left with approximately 3.16 million well-formed samples (54.1% of the original corpus). This resulting clean QUACC corpus is a balanced question answer corpus with 43 different question types. A list of the 28 most frequent question types and their percentage distribution in both the unclean and the clean QUACC data set are shown in Table 1 (the remaining 15 question types occur with less than 0.1% in both data sets).

**Table 1:** Types and percentage of question phrases in QUACC data sets.

**Question phrase**	**Clean**	**Unclean**
	**QUACC**	**QUACC**
Was (“what”)	14.517	13.417
Wann (“when”)	8.4845	6.62175
Wem (“whom_*dat*_”)	7.2055	9.9115
Wen (“whom_*acc*_”)	6.09975	7.3505
Wo (“where”)	5.52325	4.42025
Womit (“with what”)	5.5075	4.35375
Worin (“where in”)	5.507	4.3765
Wer (“who”)	5.5045	4.38675
Wozu (“what for”)	5.3245	4.365
Wofür (“what for”)	5.25725	4.34525
Wobei (“where by”)	5.106	4.371
Wonach (“after what”)	4.33225	4.391
Wovon (“of what”)	3.85725	4.36175
Warum (“why”)	3.15475	2.56925
Wohin (“where to”)	2.97575	4.39025
Worüber (“about what”)	2.12775	2.677
Wodurch (“through what”)	2.034	2.146
Weswegen (“why”)	1.3345	1.01375
Worauf (“on what”)	1.3115	1.176
Woraus (“out of what”)	1.1965	2.778
Wogegen (“against what”)	1.19475	1.394
Wie (“how”)	0.972	1.23
Woran (“on what”)	0.66975	0.768
Woher (“where from”)	0.33225	0.47475
Wovor (“what for”)	0.16075	0.329
Worum (“what about”)	0.12525	0.12
Welche (“which”)	0.086	0.21625
Worunter (“under what”)	0.072	0.28075

In the following, we will use this cleaned corpus to evaluate a number of models that have been shown to provide good results for the task of QG with QA congruence. Our goal is to show that our clean QUACC data set enables different types of neural models to produce questions of even higher quality compared to the numbers that were presented in De Kuthy et al. (2020) and Kannan et al. (2021) where various models were only trained and tested on the unclean QUACC.

## 4. Experiments on QG with question answer congruence

### 4.1. Related work

#### 4.1.1. Rule-based question generation

In computational linguistics, question generation (QG) has been tackled in several, usually applied contexts, mostly focusing on English. Automatically generating questions is a challenging task involving methods such as parsing, coreference resolution, and the transformation of syntactic structures reflecting complex linguistic characteristics. A variety of QG systems were developed, often for educational purposes, e.g., assisting students in reading (Mazidi and Nielsen, 2015), vocabulary learning (Mostow et al., 2004; Brown et al., 2005), or the assessment of reading comprehension (Le et al., 2014).

The first large-scale QG approaches were rule-based (Heilman and Smith, 2010; Chali and Hasan, 2015). They relied on manually specified syntactic rules or patterns for the question formation and linguistic features such as parts-of-speech to select the appropriate question word. However, identifying and specifying the relevant characteristics and patterns requires substantial linguistic expertise and is very time-consuming, and the resulting analysis pipelines typically do not generalize and scale well to the breadth and variability of authentic data.

Much less QG research for languages other than English, such as German, exists. Many approaches are developed within other domains, as for example Gütl et al. (2011) where the focus is on the extraction of concepts from German text, reporting very little on how questions are actually constructed. To the best of our knowledge, the work by Kolditz (2015) is the only systematic exploration of the characteristics and challenges of QG for German. The rule-based QG system he implemented selects a potential answer phrase (NPs, PPs, and embedded clauses) based on a syntactic analysis of the input sentence, replaces it with an appropriate question phrase, and transforms the syntactic representation of the declarative input sentence into question form. This system was already described in more detail in Section 3 as the basis for the creation of the our own QUACC corpus.

#### 4.1.2. Neural question generation

Current research on QG is dominated by deep learning supporting a fully data-driven, end-to-end trainable approach. In the current state-of-the-art approaches, question generation is treated as a sequence-to-sequence learning problem (Sutskever et al., 2014), where an encoder network learns the latent representation of the source sentence and the decoder network generates the target question one word at a time. One of the first neural encoder-decoder model for question generation (Du et al., 2017) introduces two such models, which are provided with the source sentence and paragraph-level information that encodes the context of the generated question. Borrowing from reinforcement learning, the work by Kumar et al. (2018) introduces policy gradients along with POS tags and named entity mentions to assign task-specific rewards to the training objective. Pointer-generator networks (Gu et al., 2016; See et al., 2017) with gated self-attention have been deployed to address the problem of rare and out-of-vocabulary words and larger contexts (Zhao et al., 2018).

The neural question generation models mentioned above, and many more in this vein, primarily focus on generating questions in English and consider words to be the atomic unit of meaning. They consequently approach the representation learning and text generation tasks at the word level. This assumption does not necessarily hold for all languages, as for example Chinese, where the individual characters contain rich internal information. As a consequence, neural language models that are trained on character-level inputs have been shown to capture more salient information about morphology than their word-level counterparts (Huang et al., 2016; Marra et al., 2018). Character-aware question answering systems (Golub and He, 2016; Lukovnikov et al., 2017) have similarly been shown to be resilient to the unknown word problem. To capture and combine information about language form and meaning, Bojanowski et al. (2017) proposed treating words as bags of character n-grams to enrich word embeddings with subword information. Byte-pair encoding (Shibata et al., 1999) has seen a recent resurgence in the context of generative language models where it is employed to perform subword segmentation without the necessity of tokenization or morphological analysis. Subword-level embeddings learned with the help of this method have been shown to be competitive in many downstream NLP tasks (Sennrich et al., 2015; Heinzerling and Strube, 2018; Xu et al., 2019).

### 4.2. QG with question-answer congruence

The task of question generation with question answer congruence was introduced in De Kuthy et al. (2020). As a first baseline for this task, they trained and tested a word based model which successfully generated the envisaged questions, but had problems with unknown words. A line of successful subword and character models was trained in Kannan et al. (2021). While these models overcame the problem of unknown words, and showed good results in terms of BLEU scores, a qualitative analysis revealed problems in particular with the correct question word selection.

We will here repeat those experiments on our clean QUACC data set and compare the results to the earlier approaches. We also investigate whether and to what extent the problems reported for the unclean QUACC data were due to errors in the training data. In a second step, we present an indepth qualitative analysis and investigate the nature of the remaining errors. In addition, we also investigate the coverage of question types and how this differs between the unclean and clean data set, providing us with more insights about the particular challenges of the task.

As the starting point for the experiments on the clean QUACC data set, we build on the same basic architecture as De Kuthy et al. (2020), a word-embedding based sequence-to-sequence model (Sutskever et al., 2014) with multiplicative attention (Luong et al., 2015). This is done in order to ensure comparability of our results on the clean QUACC data set with the earlier results on the original (uncleaned) QUACC data set.

Exploring a fundamentally different neural architecture—such as using a Transformer (Vaswani et al., 2017) or a pointer-generator (Zhao et al., 2018) network—would make it more difficult to distinguish between any improvements offered exclusively by the new clean QUACC data set as training and testing input and those by the changes in architecture. We will, however, at the end of this chapter include an outlook on the use of pre-trained language models for our task, since they have been proven to be very successful for the task of QG in the context of question answering.

### 4.3. Data preparation and features

Following the method presented in De Kuthy et al. (2020), the data of the clean QUACC data were prepared in the following way for training: The (surface-form) tokens of the source sentence, their part-of-speech tags, and the span of the answer phrase were used as inputs to the model. *spaCy* (https://spacy.io) with the de_core_news_sm pre-trained model was used for tokenization, tagging, and parsing. The answer span was encoded in IOB format. All input sequences were padded with special leading and trailing tokens to indicate their beginning and end. In the encoder stage of the model, the input at each time step was the concatenation of the embeddings of the token and the POS tag, and the answer span indicator. Pretrained *fastText* embeddings (Bojanowski et al., 2017) were used to initialize the token embedding matrix, which was then frozen during training. The embedding matrix for the POS tags was randomly initialized. A fixed vocabulary was used for both input and target sequences, which is generated from 100K most frequent words in the corpus. Out-of-vocabulary (OOV) tokens were replaced with a special marker token.

Following Kannan et al. (2021), to introduce character– and subword–level tokens, an input pipeline consisting of the following steps was used: (1) UTF-8 text normalization was performed on the input sentence, (2) the normalized input sentence was parsed using *spaCy*'s de_core_news_sm model (Honnibal et al., 2020) to perform word-level tokenization and part-of-speech (POS) tagging, (3) a second tokenization pass was performed on each word token to generate character and subword tokens, and (4) each character and subword token pertaining to a given word token was assigned the latter's POS tag and the answer phrase indicator.

For character-level tokenization, each word was decomposed into a list of its component Unicode codepoints. Subword tokenization was performed with the *HuggingFace* Tokenizer library (Wolf et al., 2020). The library provides byte-pair encoding (BPE, Shibata et al., 1999) and unigram (Kudo, 2018) tokenization algorithms. BPE first constructs a baseline vocabulary with all unique symbols in a corpus. Then, merge rules that combine two symbols in the base vocabulary into a new symbol are learned iteratively until a desired final vocabulary size is reached. Conversely, unigram tokenization starts with a large initial vocabulary from which it repeatedly removes symbols that have the least effect on a loss function defined over the training data of a unigram language model. To reduce the size of the base vocabulary in both models, base symbols are directly derived from bytes rather than (all) Unicode codepoints. The library also includes the SentencePiece (Kudo and Richardson, 2018) algorithm, which processes the input as raw string sequences obviating the need for pre-tokenization.

Finally, a bidirectional LSTM was used as the recurrent unit in the encoder since, as motivated in Kannan et al. (2021), we expect the contextual information provided by the backward pass to not only enrich the sentential representation learned in the encoder but also lower the effective reduction in learnable parameters caused by the smaller vocabulary sizes of the character- and subword-level models. The per-timestep input to the encoder is the concatenation of the token embedding, POS embedding, and the answer phrase indicator. The final outputs of the encoder (hidden state, sequences, cell state) is the concatenation of the respective backward and forward layers of each output.

For the character-level models, a fixed-size vocabulary consisting of all the unique codepoints in the QA corpus was generated. Similarly, the subword tokenizers were trained on the entire corpus to generate vocabularies with 10K symbols each^3^.

### 4.4. Training

We implemented a Seq2Seq model with multiplicative attention (Luong et al., 2015) using *TensorFlow* 2.0 (Abadi et al., 2015), with our code available upon request.

The QUACC corpus introduced in Section 3 was iteratively undersampled to create multiple sets of training, validation, and test data for different sample sizes with the same distribution of question types. We each trained versions of the model on 400K samples sets from the clean QUACC corpus. Validation data sets of 15K samples were used for all models. Teacher forcing was enabled to ensure training stability.

**Figure 4:**
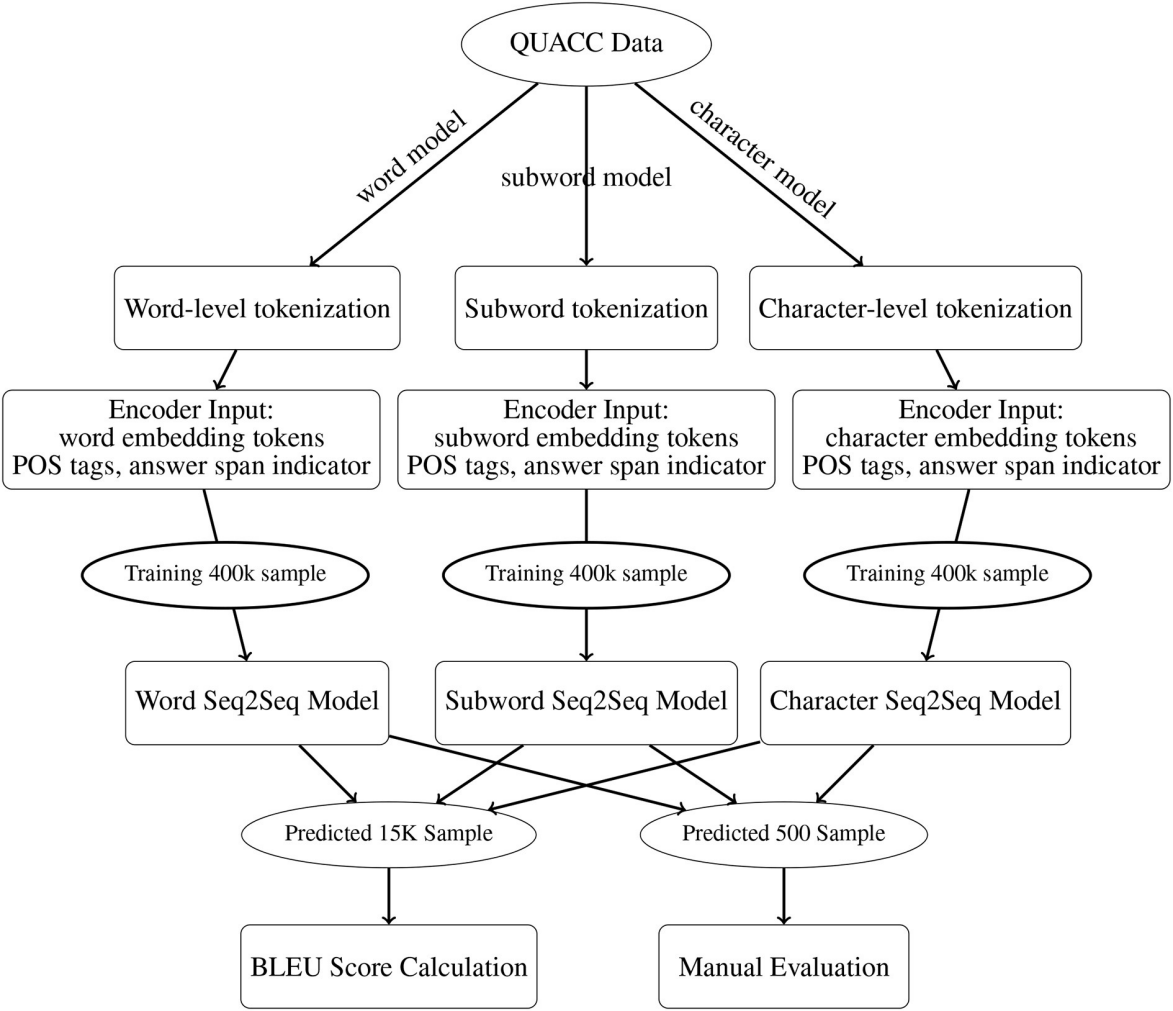
Question generation and evaluation pipeline.

For a comprehensive comparison, we trained five models: a word-level model to replicate De Kuthy et al. (2020), a subword model with one tokenization algorithms (SentencePiece Unigram), and a character model, and the same subword and character model enriched with POS features. All models were trained on the same 400K training samples from the clean QUACC corpus for 20 epochs, and validation was performed on 40K samples. For each type of input representation, the model with the lowest validation loss was evaluated on a held-out test set of 15K samples. An overview of the complete preprocessing, training and evaluation pipeline is shown in Figure 4.

Overall, the following model hyperparameters were used: batch size: 128, encoder: Bi-LSTM, decoder: LSTM, encoder/decoder hidden size: 256/512, encoder/decoder dropout: 0.5, word/subword/character embedding dim: 300, decoder beam search width: 5.

## 5. Results and evaluation

### 5.1. Quantitative results

In this comprehensive evaluation, the trained models predicted the questions for 14.700 previously-unseen sentences from both the unclean QUACC data and the clean QUACC data set and the results are compared to gold-standard questions. For this evaluation on a large test set, for which no manually validated gold-standard questions are available, we used the questions generated by the rule-based approach as the gold standard. The questions generated by the models trained on the unclean data set are thus compared to the rule-based questions from that unclean data set, and the questions from the models trained on the clean QUACC data set are compared only to the gold questions from the clean data set.

For their original model, De Kuthy et al. (2020) implemented a post-processing copy module to replace OOV marker tokens in the generated question with the original tokens from the source sentence; this behavior was replicated for the basic word-level model.

As measure we used the BLEU metric (Papineni et al., 2002) standardly employed in current QG research. The *SacreBLEU* (Post, 2018) Python library (V. 1.4.10 with default parameters) was used to calculate the cumulative and individual *n*-gram precision scores. Table 2 shows the BLEU scores from comparing the ground-truth questions of the test set with corresponding model-generated questions (best results are shown in bold).

**Table 2:** Quantitative evaluation results.

**Model**	**Features**	**BLEU unclean**	**BLEU clean**
Word + Copy	Word, Ans, POS	84.20	86.89
Subword (SentPiece Unigram)	Subword, Ans, POS	**91.76**	**94.79**
Character	Char, Ans, POS	90.18	91.18
Subword (SentPiece Unigram)	Subword, Ans	90.84	94.58
Character	Char, Ans	90.34	92.66

The BLEU scores reported for the Word model from De Kuthy et al. (2020) are the scores after applying the post-processing step to the generated questions. As reported in Kannan et al. (2021), the character- and subword-level models, on the other hand, are able to sidestep this issue by generating the target sequence one character or subword at a time. We report BLEU scores for trained variants of the character- and subword-level models without POS tags (the NoPOS models in the table). Even with fewer learnable parameters and without the linguistic information provided by the POS tags, the models are able to achieve scores very close to those of their POS-aware counterparts.

The original models trained on the unclean QUACC data set already produced very high BLEU scores, showing a very high overlap between the gold (rule-based) questions in the testing data set and the generated questions. The models trained on the clean data set show even higher BLEU score, with an improvement around 2% for all models on average. This shows that our cleaned data set enabled the models to even better learn the patterns for successful question generation.

To see whether this improved behavior is also reflected in the well-formedness of the generated questions, we now turn a qualitative investigation in terms of human evaluation and error annotation.

### 5.2. Human evaluation

To analyze the quality of the results produced by our models and compare them to those of the baseline word-level model, we performed a manual evaluation of the questions generated for the same sample of 500 sentences of De Kuthy et al. (2020).

The quality of the generated questions was manually evaluated by two human annotators, both trained linguists and native speakers of German. They were asked to provide a binary judgment: whether the question is well-formed and satisfies question-answer congruence with the source sentence. The two criteria were expressed in an annotation manual as follows:

(i) Well-Formedness: Is the question grammatically correct and would I formulate it that way as a native speaker of German? and
(ii) Question-Answer Congruence: Is the question answered by the associated sentence as a whole?

The guidelines instructed the annotators to take into account all aspects of grammaticality, including word order, verb forms, punctuation, and also spelling and capitalization errors. For the evaluation of question-answer congruence, it had to be checked whether the generated question was answerable by the full source sentence, in particular whether the question word matched the given answer phrase and whether the question did not contain any semantically different words. The resulting annotation on a small test sample of 100 question answer pairs showed good inter-annotator agreement (κ = 0.74).

The Table 3 shows the percentages of well-formed questions produced by the original 5 neural models trained on the uncleaned QUACC data vs. the models trained on the clean QUACC data (best results are shown in bold).

**Table 3:** Results per question for the evaluation set of 500 QA pairs.

**Model**	**Well-formed questions**	**Well-formed questions**
	**unclean QUACC (%)**	**clean QUACC (%)**
Word	54.2	62.2
Subword	59.6	**66.4**
(SentPiece Unigram)		
Subword	61.0	65.6
(SentPiece Unigram no POS)		
Character	**61.4**	62.4
Character	59.6	55.8
(no POS)	

Four of the five neural QG models trained on the cleaned QUACC data show major improvements in terms of number of well-formed questions over the original models trained on the uncleaned QUACC data. The word model shows the biggest improvement with 8% more well-formed questions when trained on cleaned data, followed by the subword model trained with POS features. The character model train with POS features shows the smallest improvement and the same model trained without POS features even produced less well-formed questions when trained on the clean QUACC data set.

Following the approach introduced in De Kuthy et al. (2020), we also performed a systematic error analysis of the most frequent errors to investigate where in particular the two versions of models improved.

### 5.3. Qualitative analysis—Types of errors

The purpose of this systematic error analysis is to gain more insights into how well-suited the clean QUACC data set is for the task of generating questions with question answer congruence. The results of the systematic error analysis of the most frequently encountered errors for all our models is presented in Tables 4, 5. The overall sums differ slightly from the percentages in Table 3 since one question can contain multiple types of errors.

**Table 4:** Distribution of error types in the 500 samples for models trained on unclean QUACC.

**Error type**	**Word**	**Subword**	**Subword**	**Character**	**Character**
	**POS (%)**	**POS (%)**	**no POS (%)**	**POS (%)**	**no POS (%)**
Question word	16.4	21.4	20	21.8	23.4
Unknown word	7	–	–	–	–
Different word	7	3.2	1	0.2	–
Different subword	–	0.2	0.4	–	–
Missing word	0.4	1.6	2	1.4	0.8
Missing subword	–	–	0.4	–	–
Repeated word	0.8	0.8	0.8	2	1
Word order	5.8	4	4.6	4.2	4.6
Verb form	1.6	1.8	3	2.6	3.4
Spelling	–	0.6	0.4	–	0.8

**Table 5:** Distribution of error types in the 500 sample for models trained on clean QUACC.

**Error type**	**Word**	**Subword**	**Subword**	**Character**	**Character**
	**POS (%)**	**POS (%)**	**no POS (%)**	**POS (%)**	**no POS (%)**
Question word	10	15.4	13.6	17.4	25.6
Unknown word	7.4	–	–	–
Different word	8.4	2	2.2	0.2	–
Different subword	–	0.8	0.4	–	–
Missing word	0.8	0.8	2.2	2.8	0.8
Missing subword	–	–	0.4	–	–
Repeated word	0.8	3	3	4.1	2.2
Word order	1.4	1.6	1.4	2.4	2.2
Verb form	0.8	2.6	2.6	3	4.6
Spelling	0.2	–	1.2	–	0.8

One of the problems noted in De Kuthy et al. (2020) was the occurrence of unknown words in the questions produced by the word model even after the post-processing copy mechanism. This problem still exists with a similar number of errors for the word model trained on the clean data. This is expected since the clean QUACC data very likely contain a similar percentage of rare or unknown words. Such rare words are *sffisant* (*smug*), *listenreich* (*cunning*), augenfllig (*eye-opening*), *Naschwerk* (*sweet delicacy*), *Erbtanten* (*rich aunt from which one inherits*). The subword and character models did not show this problem independently of whether being trained on the clean or the unclean QUACC data.

Another error already reported by De Kuthy et al. (2020) are unwanted word replacements with different words that occur with the word model, for example, *unbegreiflich* (*incomprehensible*) was replaced by *geschehen* (*happen*), *Adelheid Streidel* (proper name of a terrorist) by *extremistischen Streidel* (*extremist Streidel*), and *bewilligt* (*approved*) by *beantragt* (*requested*). This error occurs with a similar percentage for the word model trained on the clean data set (8.4 vs. 7%). The subword models reduce this to as few as five occurrences, and in the character models this type of error does not occur at all. This type of error, since it is not related to well-formedness of questions, also occurs with similar percentages for the models trained on the clean QUACC data, as shown by the numbers in Table 5. By far the biggest error source for all models is the production of incorrect question words. This is a hard objective since the question word depends on aspects of form (e.g., does it refer to a nominal phrase or a prepositional phrase) and meaning (e.g., does it refer to an animate or inanimate referent) of the given answer phrase. The word-level model had fewer problems with question word generation than the other models, so the word embeddings encode sufficient form and meaning information for the model to learn the question word patterns. The models variants trained on the clean QUACC data set (with the exception of the one character model) all improved to a great degree on this aspect and now only produce incorrect question word between 13 and 17% of time. This shows that cleaning the QUACC data set apparently had the intended effect of improving the QA congruence in the data and the clean data set is now a better training and testing resource for the task of QG with question answer congruence. In a similar vain, the form related error word order did occur in much smaller numbers in the questions produced by the clean model variants, showing that the clean QUACC data contain more consistent word order patterns that helped the models to produce well-formed questions. Since the other error types, like missing words or repeated words, are not related to well-formedness or QA congruence, the error numbers do not differ between the two variants of each model.

### 5.4. Qualitative analysis—Question types

The main objective for the creation of the QUACC data set was to create a data set with question answer pairs that show strict question answer congruence. Such question answer congruence only exists when the correct question word is chosen in the question. There might, however, be certain question types for which it is more difficult to learn the correct patterns than for others.

We here provide a more in-depth analysis of the question types produced by our models for the unclean and for the clean QUACC data sets. An analysis in terms of question types occurring in the questions produced for the unclean QUACC data vs. the clean QUACC data revealed that the questions from the clean data set are more balanced with respect to distribution over question types. An example analysis of number of question types, number of well-formed questions for this question type and number of question word errors for this question type is presented in Table 6 for the question sample produced by the word model.

**Table 6:** Types and frequency of question phrases in well-formed questions of the 500 sample.

	***Word unclean** **quacc***			***Word clean** **quacc***		

**Question phrase**	**Total**	**Well-formed**	**q word**	**Total**	**Well-formed**	**q word**
	**number**		**error**	**number**		**errsor**
Was (“what”)	235	124	40	182	119	18
Wer (“who”)	88	66	1	148	99	7
Wo (“where”)	19	12	1	21	14	0
Wann (“when”)	15	9	0	18	12	0
Worin (“where in”)	23	10	4	15	11	1
Wen (“whom_*acc*_”)	11	6	2	14	6	4
Wozu (“what for”)	10	6	3	10	6	3
Wovon (“of what”)	9	6	0	9	6	0
Wem (“whom_*dat*_”)	20	2	15	8	2	4
Woran (“on what”)	6	2	1	8	2	1
Worauf (“on what”)	4	3	1	8	4	3
Wobei (“where by”)	8	5	1	7	3	1
Wohin (“where to”)	7	3	1	7	3	1
Womit (“with what”)	7	3	0	7	4	0
Wofür (“what for”)	6	1	3	4	1	2
Für wen (“for whom”)	3	2	1	4	3	0
Wie lange (“how long”)	1	0	1	4	1	2
Wie oft (“how often”)	1	0	1	0	0	0
Wie weit (“how long”)	1	0	0	0	0	0
Wonach (“after what”)	4	2	1	3	1	0
Warum (“why”)	3	1	0	3	2	0
Mit wem (“with whom”)	2	1	1	2	1	1
Seit wann (“since when”)	2	2	0	2	1	0
Um was (“what about”)	2	1	0	2	1	0
Zu wem (“to whom”)	1	0	0	2	2	0
Unter wem (“under whom”)	2	0	1	1	0	0
Bei wem (“by whom”)	1	1	0	1	1	0
Laut was (“according to what”)	1	0	0	1	0	0
Von wem (“of whom”)	1	0	0	1	1	0
Wodurch (“through what”)	1	0	0	1	0	0
Wogegen (“against what”)	1	1	0	1	1	0
Woher (“where from”)	1	0	0	1	0	1
Woraus (“out of what”)	1	0	1	1	0	1
Worüber (“about what”)	1	1	0	1	1	0
Wovor (“what for”)	1	1	0	1	1	0
In wen (“in whom”)	0	0	0	1	0	0
Worunter (“under what”)	0	0	0	1	1	0
In wem (“in whom”)	1	0	0	0	0	0

The word model trained on the clean QUACC data produced questions of 35 different question types, the word model trained on the unclean QUACC data produced 37 different question types. But if one looks at the number of question types for which the models actually produced well-formed questions, then the number is down to 25 question types for both the unclean model version and 26 question types for the clean model. As discussed in Section 3, both, the clean and the unclean QUACC data sets, contain a high percentage of questions starting with *was* (“what”). The word model trained on the unclean QUACC data set produced a large number of *was* questions—235, i.e., almost half of the questions in the 500 sample set, were question starting with this question word, of which again almost half are not well-formed. The word model trained on the clean QUACC data produced less *was* questions, for which the proportion of well-formed questions then was much higher. There are other question types, as for example questions starting with *wem* (“whom_*dat*_”), for which the word model trained on the uncleaned data produced a relatively high number of questions (20) out which only 2 are well-formed and 15 wh-word error occurred in the not well-formed ones. This shows that the word model could not really pick up the correct pattern for *wem* questions from the unclean QUACC data set. The word model trained on the clean QUACC data did not attempt to produce that many *wem* questions anymore. It still only produced 2 well-formed questions starting with *wem*, but the better ratio between total number of questions and number of well-formed questions at least shows that the cleaned data set helped the model to learn when not to produce a *wem* question. This trend can also be seen for several other question words. This shows that the clean QUACC data set is a better suited data set for the task of question generation with question answer congruence where the selection of correct question types is of special importance. Similar tables with numbers of question types for the other neural models that were trained and tested on both QUACC data sets can be found in the Appendix.

Even with the clean QUACC data set, generating questions with question answer congruence, i.e., questions with the correct question word, still remains the biggest challenge for the neural question generation approaches. We here show two examples illustrating this particular challenge. In example (6) the majority of neural models trained on the unclean QUACC data produced the question word *wohin* in the first question instead of the correct question word *worauf* shown in the second question.


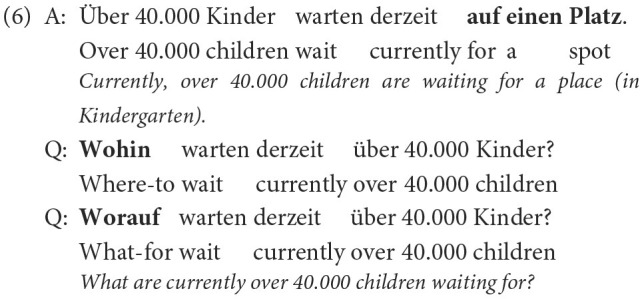


While, for example, the word model trained on the unclean data produced the incorrect *wohin*, the same model trained on the clean QUACC data produced the correct question word *worauf* , but it nevertheless produced errors with the question word *worauf* , as can be seen in Table 6. In general, a prepositional phrase starting with the preposition *auf* (“on”) can be used to indicate a direction. In that case, the question word *wohin* would be the correct one in a question. Apparently, the neural models do not learn to distinguish the various usages of the preposition *auf* in order to then generate the correct question word.

One particular challenge for the character based models is that these purely form based approaches can produce character strings that do not represent any word in the given language. This is expected to occur especially for those forms that have to be predicted (and cannot be copied over from the input), in our case for the question words. And indeed, the character models did produce non-existing question words. the question words *wott, woh, wor* were produced for questions in the 500 evaluation set.

### 5.5. Outlook on other architectures for QG

We have shown in the previous section that a number of different neural architectures can master the task of question generation tailored toward question answer congruence. But all of the models have their specific problems with the task, in particular it is difficult to generate appropriate question words to ensure the required QA congruence. We have established that our clean QUACC data set helps these models to overcome some of these problems, and we are therefore now in the position to experiment with other types of neural architectures that have been shown to be successful for different tasks in the domain of natural language generation.

Pre-trained language models have shown to be very successful for various language generation tasks (Chan and Fan, 2019; Varanasi et al., 2020). We therefore conducted first experiments with a BERT language model (Devlin et al., 2018) and explored how to successfully fine-tune this architecture using weak supervision to generate questions that satisfy question-answer congruence. The first results partially outperform the best results for this task reported in this article. Comparing the widely employed BLEU scores obtained for the generated questions with a human gold standard evaluation, we experienced the limits of measures such as BLEU for assessing highly performing question generation models: The BLEU scores were in a similar range as the highest BLEU scores for the subword models reported above, while the human evaluation showed a great improvement in the quality of the generated questions over the seq2seq models discussed here. We therefore believe that in order to successfully explore pre-trained language models for our task of question generation we need a more accurate evaluation method that reflects the quality of the generated questions and can correctly measure differences between models with different parameter settings. Such methods have already been explored to a certain degree, as for example BARTScore (Yuan et al., 2021), but again first explorations of this method for the evaluation of questions generated in our context did not result in sufficient correlation with the human evaluation.

## 6. Conclusion

We established a German QA data set, that QUACC corpus which is especially designed for the evaluating methods tailored toward question generation with question-answer congruence.

We employed a rule-based question generation model to generate this large corpus of sentence-question-answer triples. The corpus was used to train and test several neural question generation models which, given a sentence and a possible answer phrase, generate the matching question. An indepth evaluation of the questions produced by these models in terms of a human evaluation including a detailed error analysis showed that the clean QUACC data enable neural models of different kinds to produce a set of questions that is more well-formed and balanced in terms of questions types compared to the set of questions produced by the same models trained on the original unclean version of the QUACC data.

## Data availability statement

The raw data supporting the conclusions of this article will be made available by the authors, without undue reservation.

## Author contributions

All authors contributed to the conception, design, and analysis of the study and experiments. All authors contributed to the manuscript revision, read, and approved the submitted version.

## Funding

The work in this paper has been funded by the Deutsche Forschungsgemeinschaft (DFG, German Research Foundation)—SFB 833—Project ID 75650358. DM is a member of the Cluster of Excellence Machine Learning—New Perspectives for Science, EXC 2064/1, project number 390727645. Aspects of the material of this manuscript have been presented in part at the 28th International Conference on Computational Linguistics, COLING 2020 (De Kuthy et al., 2020) and the 14th International Conference on Natural Language Generation, INLG 2021 (Kannan et al., 2021).

## Conflict of interest

The authors declare that the research was conducted in the absence of any commercial or financial relationships that could be construed as a potential conflict of interest.

## Publisher's note

All claims expressed in this article are solely those of the authors and do not necessarily represent those of their affiliated organizations, or those of the publisher, the editors and the reviewers. Any product that may be evaluated in this article, or claim that may be made by its manufacturer, is not guaranteed or endorsed by the publisher.

## Footnotes

^1^ https://huggingface.co/german-nlp-group/electra-base-german-uncased

^2^ https://commoncrawl.org/

^3^The subword vocabularies also include the base symbols found in the character vocabulary. In both cases, special meta tokens such as unknown, sentence-start and end markers were additionally added to each vocabulary.
